# Similar appearance of different multifocal carpal bone destructing disease entities in 3 patients: A case report

**DOI:** 10.1097/MD.0000000000026445

**Published:** 2021-07-16

**Authors:** Jun-Ku Lee, Young Woo Kwon, Jae Chan Shim, Yun Kyung Kang, Weon Min Cho, Jong Woong Park, Soo-Hong Han

**Affiliations:** a Department of Orthopedic Surgery, National Health Insurance Service Ilsan Hospital, Goyang; b Department of Orthopedic Surgery, Uijeongbu Eulji Medical Center, Uijeongbu-si Gyeonggi-do; c Department of Radiology, Sanggye Paik Hospital; d Department of Pathology, Seoul Paik Hospital, Inje University College of Medicine, Seoul; e Department of Orthopedic Surgery, CHA Bundang Medical Center, CHA University School of Medicine, Seongnam-si, Gyeonggi-do; f Department of Orthopedic Surgery, Korea University Anam Hospital, Seoul, South Korea.

**Keywords:** calcifying aponeurotic fibroma, osteolysis, rheumatoid arthritis, tenosynovial giant cell tumor, wrist joint

## Abstract

**Rationale:**

Several diseases feature tumors, or tumor-mimicking lesions, that further invade the bone and surrounding joints of the wrist region. Here, we describe 3 rare cases of multiple destructed carpal bones and adjacent joints in different disease entities confirmed via pathologic diagnosis.

**Patient concerns:**

All 3 cases were examined between January 2016 and December 2019. Three patients presented with similar clinical manifestations and radiographic features, with multiple osteolytic lesions in the carpal bones and metacarpal bone base.

**Diagnoses:**

The 3 cases were diagnosed as diffuse type tenosynovial giant cell tumor, calcifying aponeurotic fibroma, and rheumatoid arthritis.

**Interventions:**

Separate, experienced radiologist and pathologist took part in the interpretation and compartmentalization of radiographs and pathological findings, respectively. Even magnetic resonance imaging could not achieve a diagnosis; surgical excision was therefore required, with subsequent pathological assessment for treatment and final diagnosis.

**Outcomes:**

functional outcomes also differed among patients, poorest in rheumatoid arthritis patient.

**lessons:**

We report 3 rare disease entities, presenting with multifocal osteolytic lesions in the wrist. They all presented with similar clinical manifestations, and the final diagnoses were made via pathological evaluation. Compared with tenosynovial giant cell tumor and calcifying aponeurotic fibroma, rheumatoid arthritis had the poorest outcome.

## 1 Introduction

The wrist is a complex and composite joint, formed by 20 interdependent articulations that bind 15 bones from the distal radioulnar joint, to the proximal parts of the metacarpus.^[1]^ Several diseases are reported to feature tumors or tumor-mimicking lesions that proceed to further invade the bone and surrounding joints in the wrist region.^[2–4]^ These conditions are uncommon and present various clinical symptoms, from being painless, to a wide array of limits regarding range of motion in practice.^[5]^

Multiple, localized, osteolytic lesions in the carpal bones are rarely seen invading adjacent joints on radiographs; moreover, there are difficulties regarding diagnosing these disease entities based on clinical symptoms and plain radiography. Even magnetic resonance imaging (MRI) could not determine the cause of bone invasion; therefore, surgical excision was eventually required, with subsequent pathological assessment for a final diagnosis.^[6–8]^

Herein, we describe 3 cases presenting with similar clinical manifestations and radiological features, including multiple destroyed carpal bones and adjacent joints, where different disease entities are confirmed via pathologic diagnosis. Our purpose was to introduce and compare these rare conditions in terms of radiology, pathology, and treatment outcome.

## 2 Case presentations

Between January 2016 and December 2019, we encountered 3 cases of multifocal osteolytic lesions on plain wrist x-ray, with similar clinical manifestations. This study was conducted with the approval of the Institutional Review Board, and all 3 patients consented to the publication of this study.

An experienced radiologist interpreted and compared the x-ray, computed tomography (CT), and MRI radiographs of the 3 patients, whereas an experienced pathologist was responsible for interpreting and comparing the pathology of the 3 patients.

### 2.1 Case 1

A 54-year-old male patient was referred by a local clinic for right wrist dorsum pain, starting 7 years before and aggravating a month before referral. The patient complained of 1∼2 abrupt pain episodes, with a pain visual analogue score (VAS) of 2 to 3; the elicit pain lasted for <30 minutes. The patient did not report any trauma or medical history.

Upon physical examination, the patient presented with mild and diffuse swelling and slight tenderness in the affected wrist dorsum, compared with the left. The range of motion was limited to volar flexion of 50 degree, and extension of 40 degree; mild pain occurred with terminal flexion and extension.

**Figure 1:**
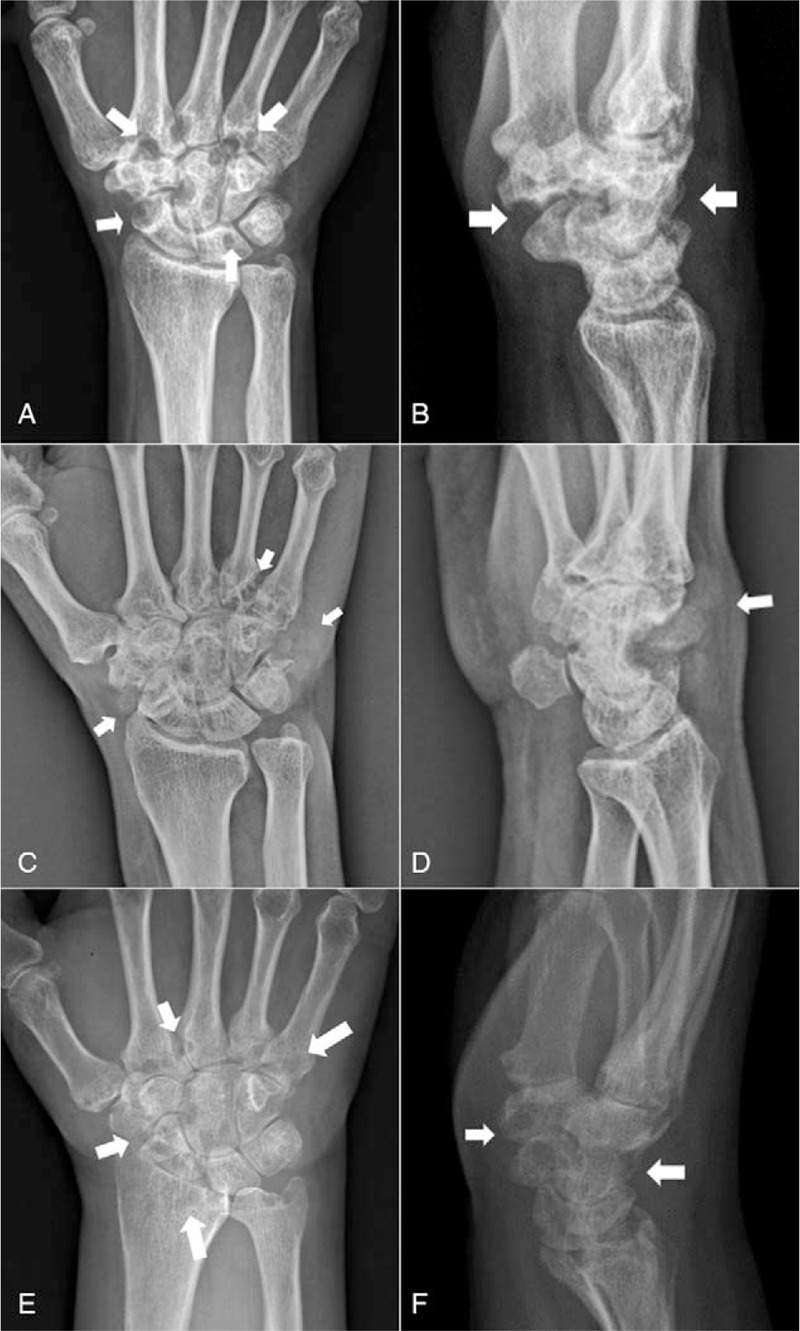
Plain x-ray, anteroposterior (AP), and lateral views of the 3 patients. Plain radiographs of Case 1 (A and B) reveal multiple well-defined carpal and metacarpal base erosions (arrows). In Case 2, the AP (C) and lateral views (D) show multiple erosions of the carpal bones and metacarpal bases, with surrounding, ill-defined, lobulated soft tissue density (arrows). Case 3 (E and F) also reveals multiple erosions of the radius, carpal bones, and metacarpal bases (arrows), with narrowing of the radiocarpal joint space.

**Figure 2:**
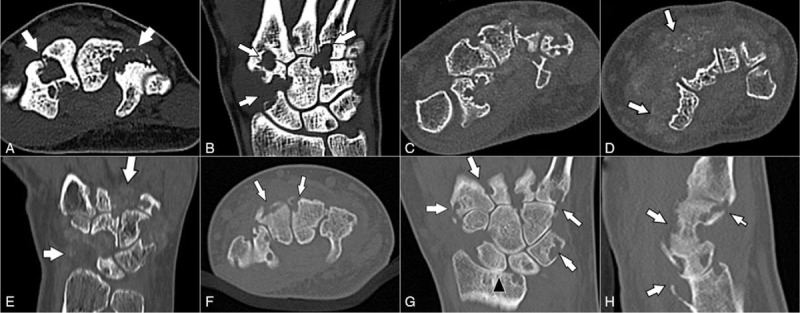
Computed tomography images of the 3 patients. In Case 1, the axial (A) and coronal (B) images reveal multiple erosions of the carpal bones and metacarpal bases (arrows). The axial (C and D) and coronal (E) images of Case 2 showed multiple erosions of the carpal bones and metacarpal bases; surrounding, ill-defined, lobulated, soft tissue mass-like lesions show stippled calcification (arrows). The axial (F), coronal (G), and sagittal (H) images of Case 3 demonstrate multiple erosions of the radius, carpal bones, and metacarpal bases (arrows), with narrowing of the radiocarpal joint space (arrowhead).

**Figure 3:**
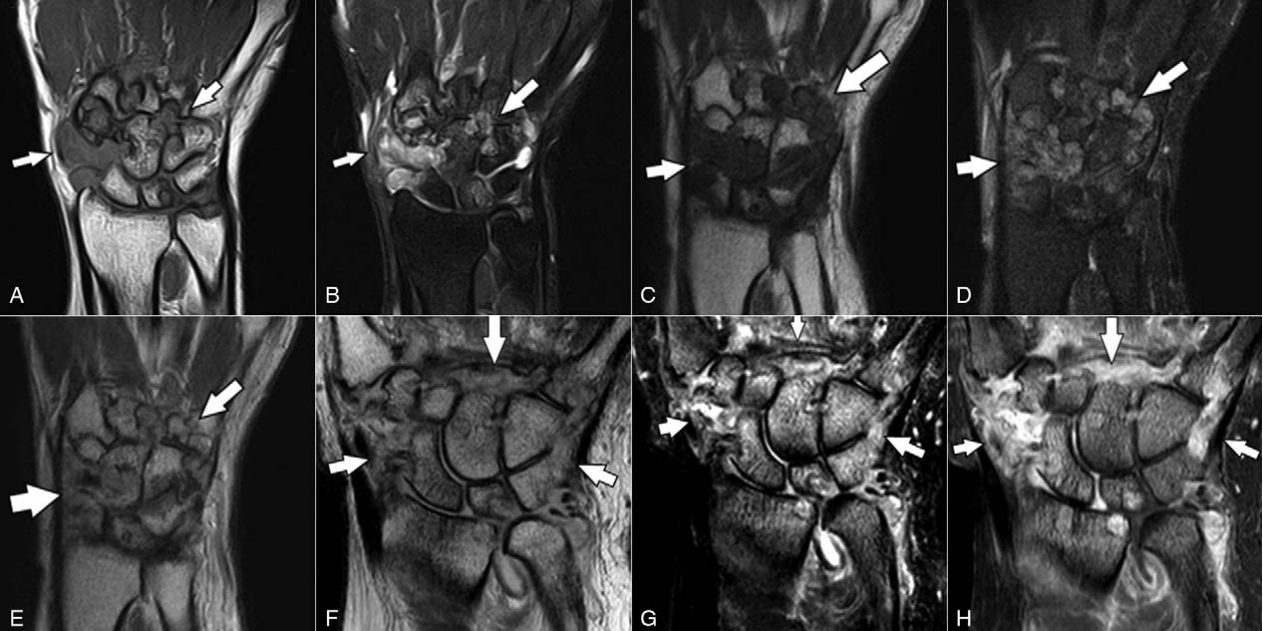
Magnetic resonance images of the 3 patients. In Case 1, multiple nodular, synovial, proliferative lesions, causing erosion of the carpal bones and metacarpal bases, revealed a low signal intensity on coronal T1-weighted imaging (A), and a heterogeneous high signal intensity on coronal fat-suppressed T2-weighted imaging (B) (arrows). In Case 2, multiple nodular, synovial, proliferative lesions, causing erosion of the carpal bones and metacarpal bases, showed a low signal intensity on coronal T1-weighted imaging (C), heterogeneous high signal intensity on coronal fat-suppressed T2-weighted imaging (D), and heterogeneous enhancement (E) (arrows). In Case 3, diffuse, synovial, proliferative lesions, causing erosion of the radius, carpal bones, and metacarpal bases demonstrated a heterogeneous high signal intensity on coronal intermediate (modified proton density-weighted) imaging (F) and fat-suppressed T2-weighted imaging (G), and heterogeneous enhancement (H) (arrows). The radiocarpal joint shows cartilage loss and joint space narrowing, with distal radioulnar compartment effusion. Diffuse bone marrow edema is seen in the distal radius and ulna, carpal bones, and metacarpal bases.

Using plain radiography for the wrist in the anteroposterior (AP) and lateral views (Fig. 1A and B), there were multiple rounds of oval, erosive lesions involving the carpal bones and distal carpometacarpal (CMC) joints; these findings were also confirmed by CT (Fig. 2A and B). MRI showed multiple nodular, synovial lesions, eroding the carpal bones and metacarpal bases (Fig. 3A and B).

Laboratory examinations were performed to rule out infection and inflammatory disease; the white blood cell (WBC) count and erythrocyte sedimentation rate (ESR), as well as C-reactive protein (CRP), uric acid, and rheumatoid factor levels, were within their normal ranges.

**Figure 4:**
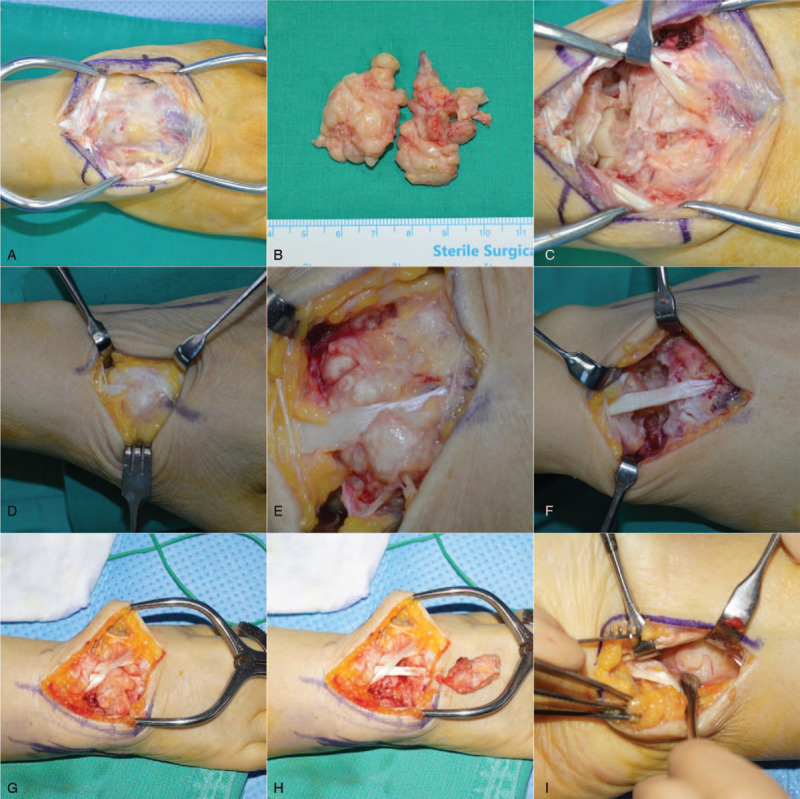
Intraoperative clinical photos of the 3 patients. In the first patient, the tumor was buried in the carpal bone and CMC joint; the articular capsule and boundary under the extensor tendon were unclear (A). Marginal excision was performed to remove all potential masses (B and C). In the second patient, the mass was found in the subcutaneous layer, enveloping the extensor tendons (D). The calcified mass invading the surrounding retinaculum and joint capsule was not distinguished (E), and was detached from the bone and adjacent joints (F). In the third patient, solid, mass-like synovium invaded the retinaculum, joint capsule, underlying bone, and joints with intact tendon (G), and was subsequently removed (H). A separated volar approach was used to remove the volar aspect lesion (I).

Marginal excision of potential lesions detected via preoperative MRI was attempted for surgery; under anesthesia, a dorsal, midline, longitudinal incision was made over the affected wrist dorsum. The mass adhered to the extensor tendon sheaths, violating the underneath capsule without distinct margination (Fig. 4A). Multiple separated, firm lesions invaded multiple carpal bones, as well as carpal, midcarpal, and CMC joints; nevertheless, the mass was easily separated from the embedded carpal bone, and the extensor tendons were intact (Fig. 4B). We attempted to extract every lesion marked on the preoperative MRI (Fig. 4C).

**Figure 5:**
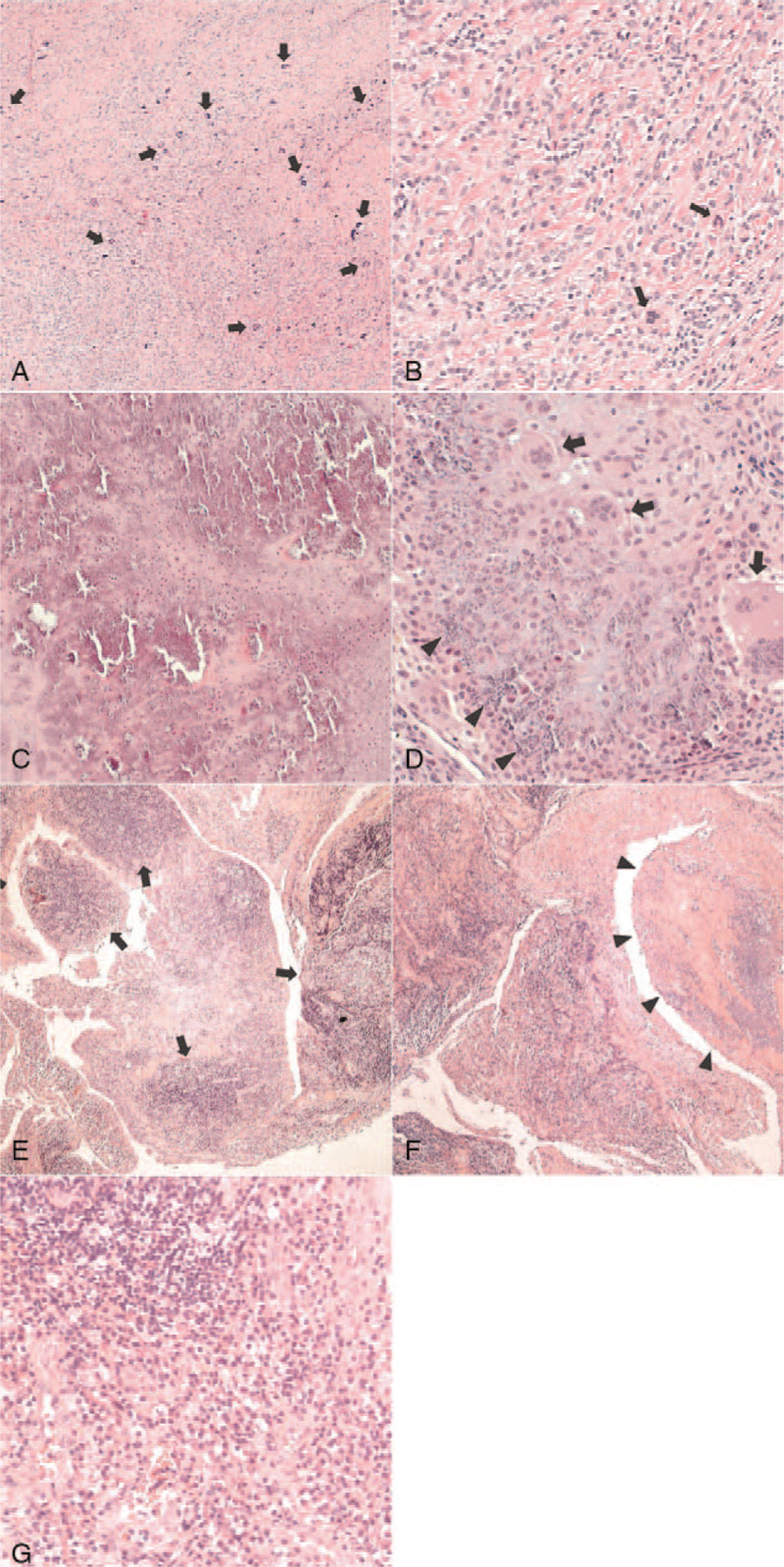
Pathological findings of the 3 patients. Case 1: giant cell tumor of the tendon sheath (A and B); Case 2: calcifying aponeurotic fibroma (C and D); Case 3; rheumatoid arthritis (E, F, and G). (A) Moderately cellular tumor showing abundant mononuclear cells and evenly scattered, multinucleated, osteoclast-like giant cells (arrows) within hyalinized fibrous stroma (HE ×40). (B) The mononuclear component of the tumor contains small to medium sized cells, with ovoid nuclei and a pale cytoplasm. Osteoclast-like giant cells (arrows) are occasionally admixed (HE ×200). (C) Zone of hyalinized chondroid matrix and chondrocyte-like cells with calcified nodules (HE ×100). (D) Cellular area showing plump/epithelioid to spindle cells, osteoclast-like giant cells (arrows), and spotty calcification (arrowheads) (HE ×200). (E) Proliferative synovitis with villous hypertrophy, and dense inflammatory cell infiltration with lymphoid follicles (arrows) (HE ×40). (F) Presence of fibrinoid necrosis (arrowheads) (HE ×40). (G) Inflammatory infiltrates are primarily composed of lymphocytes (upper left) and plasma cells (lower right) (HE ×200).

Pathological evaluation revealed moderately cellular tumors showing abundant mononuclear cells, and evenly scattered, multinucleated, osteoclast-like giant cells within the hyalinized fibrous stroma; this was consistent with tenosynovial giant cell tumors (TSGCTs) (Fig. 5A and B).

A short arm splint was applied for pain control, and the range of motion increased after a week. After 18 months of outpatient follow-up, the pre-operative symptoms disappeared, with no evidence of recurrence; however, the wrist's range of motion did not improve, with 40 degree volar flexion and 60 degree extension. The Disabilities of the Arm, Shoulder, and Hand (DASH) scores was 5.0.

### 2.2 Case 2

A 59-year-old female patient was referred by another hospital, with 2 months of mild swelling and discomfort in her left wrist, and abnormal radiographic findings; she had no history of trauma or medical history before wrist symptoms. The pain VAS was only 1 or 2, and daily activity was rarely interfered with. Upon physical examination, only mild, ill-defined swelling was found, without local heat, erythema, or even tenderness. Although the range of motion was not limited, the patient complained of discomfort when exercising full wrist flexion or extension.

Plain radiographs (Fig. 1C and D) demonstrated multiple carpal and metacarpal base erosions, surrounded by an ill-defined, lobulated, mass-like density. Preoperative CT showed stippled calcification within the surrounding mass-like lesions (Fig. 2C and D). MRI revealed multiple, heterogeneously enhancing, nodular mass-like lesions, causing well-marginated erosion of the carpal bones and metacarpal bases (Fig. 3C–E). Laboratory examinations yielded no significant results, including a normal WBC count, and ESR/CRP, rheumatoid arthritis factor, and uric acid levels.

Surgical excision was performed for exact diagnosis and treatment, and the mass was found after subcutaneous dissection of the surrounding extensor tendon (Fig. 4C). With deep dissection, the mass was buried in multiple carpal and metacarpal bones as well as surrounding joints without tendon damage. Contrasting with Case 1, the gross calcification over the protruded lesion was intraoperatively found (Fig. 4D). We also attempted to remove all possible masses identified on preoperative MRI through 2 separate longitudinal operative incisions (Fig. 4E).

Pathology was consistent with calcifying aponeurotic fibroma, confirmed by nodules of calcification with cartilaginous differentiation, osteoclast-like giant cells, and fibrotic, variably cellular stroma (Fig. 5C and D).

At the 2-year postoperative follow-up, the patient reported a pain VAS of 0 to 1, with a 4.2 DASH score and almost full range of motion in the right wrist, compared with the unaffected left side. There were no signs of recurrence, and a follow-up visit was scheduled for recurrent symptoms.

### 2.3 Case 3

A 59-year-old female presented with a year of slowly progressing right wrist pain, without any trauma or medical morbidity. On inspection, the wrist was slightly swollen, compared with the left wrist. The patient acknowledged diffuse tenderness from the radial and ulnar aspects, as well as the wrist dorsum; she noted that whenever the affected wrist was hit, a sudden and severe pain with a VAS of 8 to 9 occurred, lasting for several minutes. The range of motion was restricted to the volar and dorsal regions at 70 degree.

Plain radiographs revealed multiple erosions at the radius, carpal bones, and metacarpal bases, with narrowing of the radiocarpal joint space (Fig. 1E and F); these were clearly observed on the CT (Fig. 2F–H) scans. MRI revealed multiple erosions of the radius, carpal bones, and metacarpal bases, caused by diffuse, synovial, proliferative lesions, loss of radiocarpal joint cartilage, and narrowing of joint space (Fig. 3F–H). The preoperative laboratory findings were as follows: elevated ESR: 69 mm/h; rheumatoid factor: 20.5 IU/mL; and positive anticyclic citrullinated peptide antibody: 124 U/mL. Other laboratory findings were within the normal ranges, including complete blood cell count, as well as uric acid and CRP levels. Although rheumatoid arthritis was suspected, the tenosynovectomy with subsequent biopsy was planned considering patient's severe clinical symptoms and aggressive radiologic feature.

A longitudinal, vertical incision was made over the center of the dorsal wrist. The extensor tendons were intact; however, the dorsal capsule was indistinct, with firm, mass-like lesions, embedded within the carpal bone and joints (Fig. 4G). The lesions invaded the distal radiocarpal joint, radial-ulnar joint, dorsal aspect of the distal ulnar head, and even the volar aspect of the distal radiolunate joint (Fig. 4H and I).

Pathological examination revealed proliferative synovitis with villous hypertrophy and fibrinoid necrosis, extensive lymphoplasmacytic and histiocytic infiltration, and lymphoid follicle/germinal center formation, indicating rheumatoid arthritis (Fig. 5. E–G). The patient was referred to the rheumatologic department for subsequent medical management.

At the final outpatient follow-up at 12 months post-operation, the patient's clinical symptoms improved, with a pain VAS of 1 to 2, and DASH score of 17.2. As indicated using plain radiography, the osteoarthritis did not progress; however, the range of motion was further limited, with 30 degree volar flexion and 50 degree extension, but full pronation and supination.

Comparisons between the 3 patients, especially on radiographs, are depicted in Table 1.

**Table 1:** Comparison among 3 patients (Case 1; giant cell tumor of tendon sheath/Case 2: calcifying aponeurotic fibroma/Case 3 rheumatoid arthritis).

	Case 1	Case 2	Case 3
Age/sex	54/Man	59/Woman	59/Woman
Radiograms			
Bony invasion			
Metacarpal bases	+	+	+
Distal carpal bones	+	+	+
Proximal carpal bones	+	+	+
Distal radius or ulna	−	−	+
Joints involvement			
Carpometacarpal	+	+	+
Midcarpal	+	+	+
Radiocarpal	−	−	+
Other findings			
Volar side combined	−	+	++
Calcification	−	+	−
Arthritis	−	−	+
Tendon injury	−	−	−

## 3 Discussion

We report 3 cases of multiple osteolytic lesions in the carpal bones and metacarpal bone base, with similar clinical manifestations, treated via surgical excision. Different diagnoses were obtained, and the results also differed.

The 3 patients were diagnosed with TSGCT (Case 1), calcifying aponeurotic fibroma (Case 2), and rheumatoid arthritis (Case 3); each case presented with traits distinct from their common disease characteristics.

TSGCTs, the second most common soft tissue tumor of the hand, arise from the synovial cells of the tendon sheath, commonly occurring on the volar surface of the first 3 fingers.^[5]^ There are 2 types of TSGCT: localized and diffuse; the latter type is less frequent and more aggressive. Given that multiple lesions were invading the bones, accompanied by pain, this case was of the diffuse type. Hand dorsum occurrence, combined with multiple bone and intra-articular invasions, was a very rare incidence among TSGCTs.^[9,10]^

Calcifying aponeurotic fibroma, also known as juvenile aponeurotic fibroma, is a rare, slow-growing, painless, and benign soft tissue tumor. It also has features of local surrounding tissue invasion, but rarely involves bone destruction, typically occurring in the palm of the hand and soles of the feet in children and adolescents, involving the deep volar fascia, tendons, and Apo neuroses of the hands, as well as those of the feet.^[4,5]^ Our second patient was diagnosed with this disease, which occurred primarily in the wrist dorsum, combined with multiple bony destructions. During the study period, we found that this particular case was already reported in 2017 by another department working at one of the authors’ institutions.^[3]^

Rheumatoid arthritis is a disease of the synovium; dorsal wrist synovitis, containing the extensor tendon, is a commonly involved lesion.^[11]^ Dorsal tenosynovitis, characterized by swelling of the wrist dorsum, may be the first sign of rheumatoid arthritis. Our third patient, diagnosed with RA, had no family or medical history of RA; additionally, no other joints were affected. Usually, isolated dorsal tenosynovitis is painless; therefore, one should look for involvement of the radiocarpal or radioulnar joints, with developing pain, as observed in our patient.^[1]^ The initially thin, fluid like synovium becomes thickened, taking on a more solid appearance often confused for tumor lesions in disease progression. However, contrasting with common tendon damage in disease progression, there was no extensor tendon partial rupture.

When comparing the x-ray and CT images between the 3 cases, the second presented with calcification that differed from that of the other patients. The third patient only exhibited associated osteoarthritic changes in the radiolunate joint, whereas the distal ulnar head's bony erosion differed from the other patient's. No other distinctive characteristics were identified among the 3 cases. On MRI comparison, the first and second patients were not distinct from one another; however, the second patient exhibited calcification and a less defined tumor margin than the first.^[4,8]^ MRI was therefore inconclusive for either disease, showing an enhanced mass lesion with gadolinium infusion. The third case was distinct in that the synovium was thickened and hypertrophied, as opposed to containing nodular lesions. Joint problems were also found, including radioulnar joint effusion and osteoarthritic changes in the radiolunate joint.

Eventually, final diagnoses were reached through the pathology results. All 3 patients were treated by surgically excising the masses and solid synovium. The patient with calcifying aponeurotic fibroma exhibited the mildest preoperative symptoms and best postoperative results; conversely, the RA patient exhibited the worst pre- and postoperative results.

There have been reports of recurrence, even after removing detected lesions, in about 5–50%^[12,13]^ and 50%^[14]^ of diffuse type TSGCTs and calcifying aponeurotic fibroma cases, respectively. Fortunately, after a year of follow-up, no symptom recurrence was observed in either the TSGCT or calcifying aponeurotic fibroma cases, based on patients’ clinical symptom resolution and improved functional score; however, we did not conduct follow-up MRI. Additionally, the chance of recurrence remains even decades later; nevertheless, observation of a recurrent lesion is certainly an option if there is no functional impairment.^[15]^

The third patient was referred to the rheumatoid department; further medical treatment followed. Preoperative joint involvement and arthritis did not deteriorate a year after surgery; however, the final outcome was the lowest among the 3 patients, with a decreased range of motion compared with preoperative joint involvement, despite improvement of clinical symptoms.

## 4 Conclusions

We reported 3 rare disease entities, presenting with multifocal osteolytic lesions in the wrist. They all presented similar clinical manifestations, and the final diagnosis was made by pathological evaluation; RA had the poorest outcome, compared with diffuse type TSGCT and calcifying aponeurotic fibroma.

## Author contributions

All authors have read and approved the manuscript

**Commented on and revised the manuscript draft for critical content and approved the final version:** Jong Woong Park, Soo-Hong Han.

**Conceptualization:** Jong Woong Park.

**Data curation:** Young Woo Kwon, Yun Kyung Kang.

**Data entry, checking, recoding, analysis:** Jun-Ku Lee, KYO, Weon Min Cho.

**Formal analysis:** Weon Min Cho.

**Investigation:** Young Woo Kwon, Jae Chan Shim, Yun Kyung Kang, Weon Min Cho.

**Methodology:** Jun-Ku Lee, Jae Chan Shim.

**Pathologic resulting and interpretation:** Yun Kyung Kang.

**Project administration:** Weon Min Cho.

**Radiologic resulting and analysis:** Jae Chan Shim.

**Resources:** Weon Min Cho, Jong Woong Park.

**Supervision:** Jong Woong Park, Soo-Hong Han.

**Validation:** Yun Kyung Kang, Jong Woong Park, Soo-Hong Han.

**Writing – original draft:** Jun-Ku Lee.

**Writing – review & editing:** Soo-Hong Han.

**Wrote first draft of manuscript:** Jun-Ku Lee.

**Wrote protocol:** Jun-Ku Lee, Soo-Hong Han, Jong Woong Park.
